# Bis[2,6-bis­(benzimidazol-2-yl)pyridine-κ^3^ *N*,*N*′,*N*′′]nickel(II) bis­(tri­fluoro­methane­sulfonate) diethyl ether monosolvate

**DOI:** 10.1107/S2414314624000889

**Published:** 2024-01-31

**Authors:** Sarah E. Ruiz, Hadi D. Arman, Rafael A. Adrian

**Affiliations:** a Department of Chemistry and Biochemistry, University of the Incarnate Word, San Antonio, Texas 78209, USA; b Department of Chemistry, The University of Texas at San Antonio, San Antonio, Texas 78249, USA; Katholieke Universiteit Leuven, Belgium

**Keywords:** crystal structure, nickel(II), tri­fluoro­methane­sulfonate salt, 2,6-bis­(2-benzimidazol­yl)pyridine, octa­hedral geometry, hydrogen bond, diethyl ether solvate

## Abstract

In the title complex, [Ni(C_19_H_13_N_5_)_2_](CF_3_SO_3_)_2_·(CH_3_CH_2_)_2_O, the central Ni^II^ atom is sixfold coordinated by three nitro­gen atoms of each 2,6-bis­(2-benzimidazol­yl)pyridine ligand in a distorted octa­hedral geometry with two tri­fluoro­methane­sulfonate ions and a mol­ecule of diethyl ether completing the outer coordination sphere of the complex. Hydrogen bonding contributes to the organization of the asymmetric units in columns along the *a* axis generating a porous supra­molecular structure. The structure was refined as a two-component twin with a refined BASF value of 0.4104 (13).

## Abstract 2

In the crystal structure of the title compound, the nickel(II) metal center is surrounded by two tridentate 2,6-bis­(2-benzimidazol­yl)pyridine ligands in a distorted octa­hedral geometry. Hydrogen bonding is present between counter-ion and ligand.

## Graphical abstract

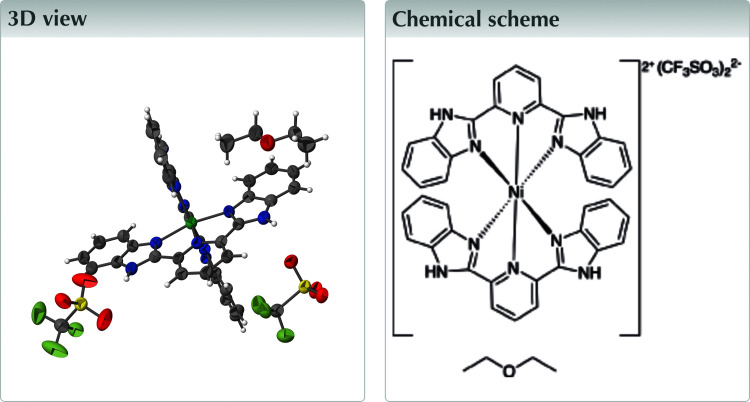

## Structure description

**Figure 1:**
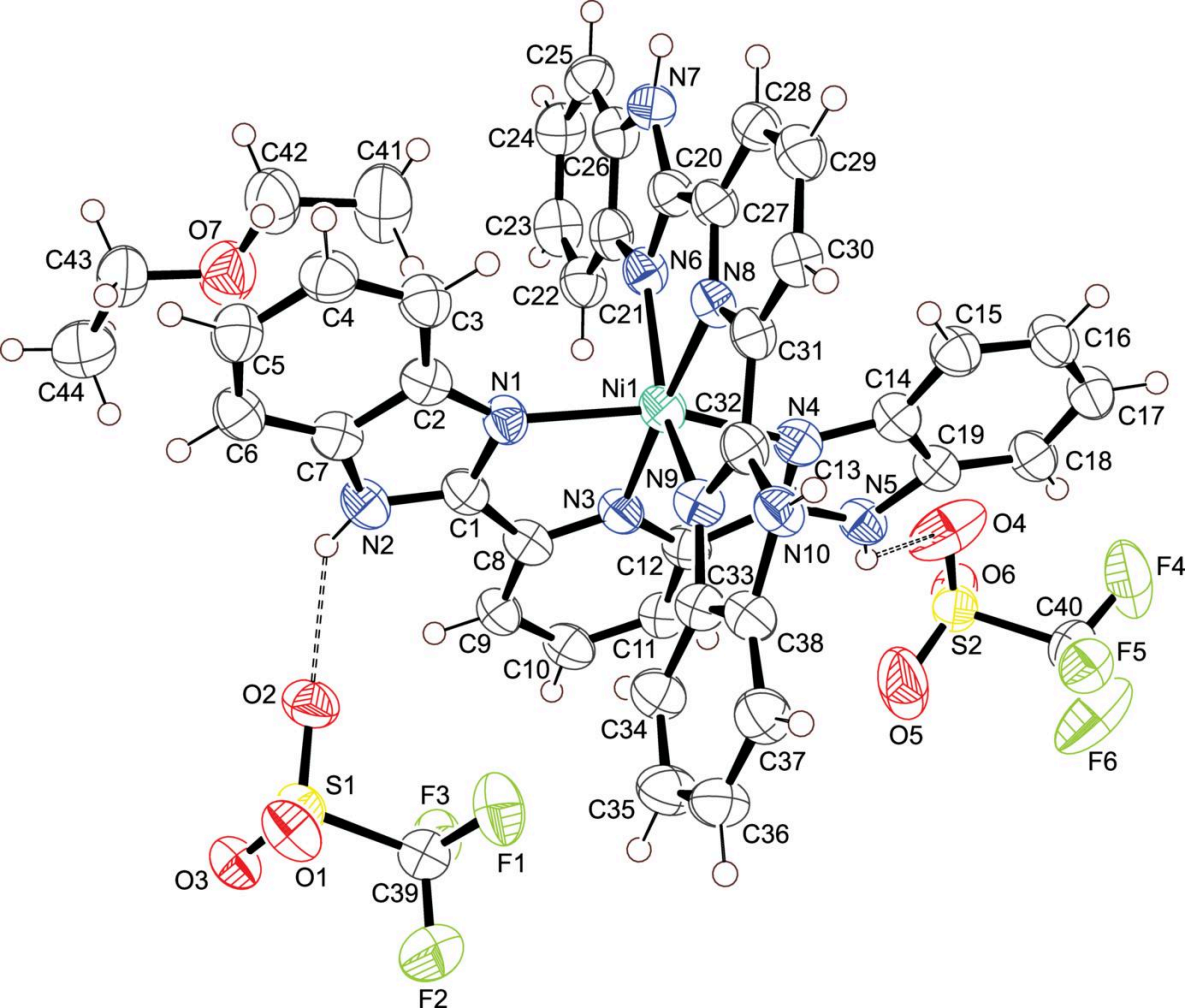
Asymmetric unit of the title compound with displacement ellipsoids drawn at the 50% probability level; Hydrogen bonds are shown as dashed lines.

Complexes bearing 2,6-bis­(2-benzimidazol­yl)pyridine (bbp) as a chelating ligand have garnered considerable inter­est due to their application in biological systems (Icsel *et al.*, 2020*a* ▸; Singh *et al.*, 2023 ▸; Šindelář & Kopel, 2023 ▸). Recently, a nickel(II) saccharinate 2,6-bis­(2-benzimidazol­yl)pyridine complex has shown considerable anti­cancer effects against A549 and MCF-7 cancer cells (Icsel *et al.*, 2020*b* ▸). Our research group inter­est currently lies in synthesizing metal complexes with applications in bio­logical systems; as part of our research in this area, herein, we describe the synthesis and structure of the title nickel(II) complex (Fig. 1 ▸).

The asymmetric unit only contains the title compound, with two symmetry-related entities inside each unit cell. The nickel(II) ion shows a distorted octa­hedral coordination environment defined by two bbp ligands, with two tri­fluoromethane­sulfonate ions and a diethyl ether mol­ecule in the outer coordination sphere. All the Ni—N bond lengths are in good agreement with comparable nickel(II) bbp complexes currently available in the Cambridge Structural Database (CSD, version 5.45, Nov 2023; Groom *et al.*, 2016 ▸; refcodes BEQTAV; Harvey *et al.*, 2018 ▸; DURWUJ; Huang *et al.*, 2010 ▸; MUNDAD; Ivanova *et al.*, 2020 ▸; ZOTVIP; Wei *et al.*, 2015 ▸; KUPFUZ; Icsel *et al.*, 2020*b* ▸), The N—Ni—N angles also concur with the values reported in the previously referenced nickel(II) bbp complexes. All relevant bonds and angles are presented in Table 1 ▸.

**Figure 2:**
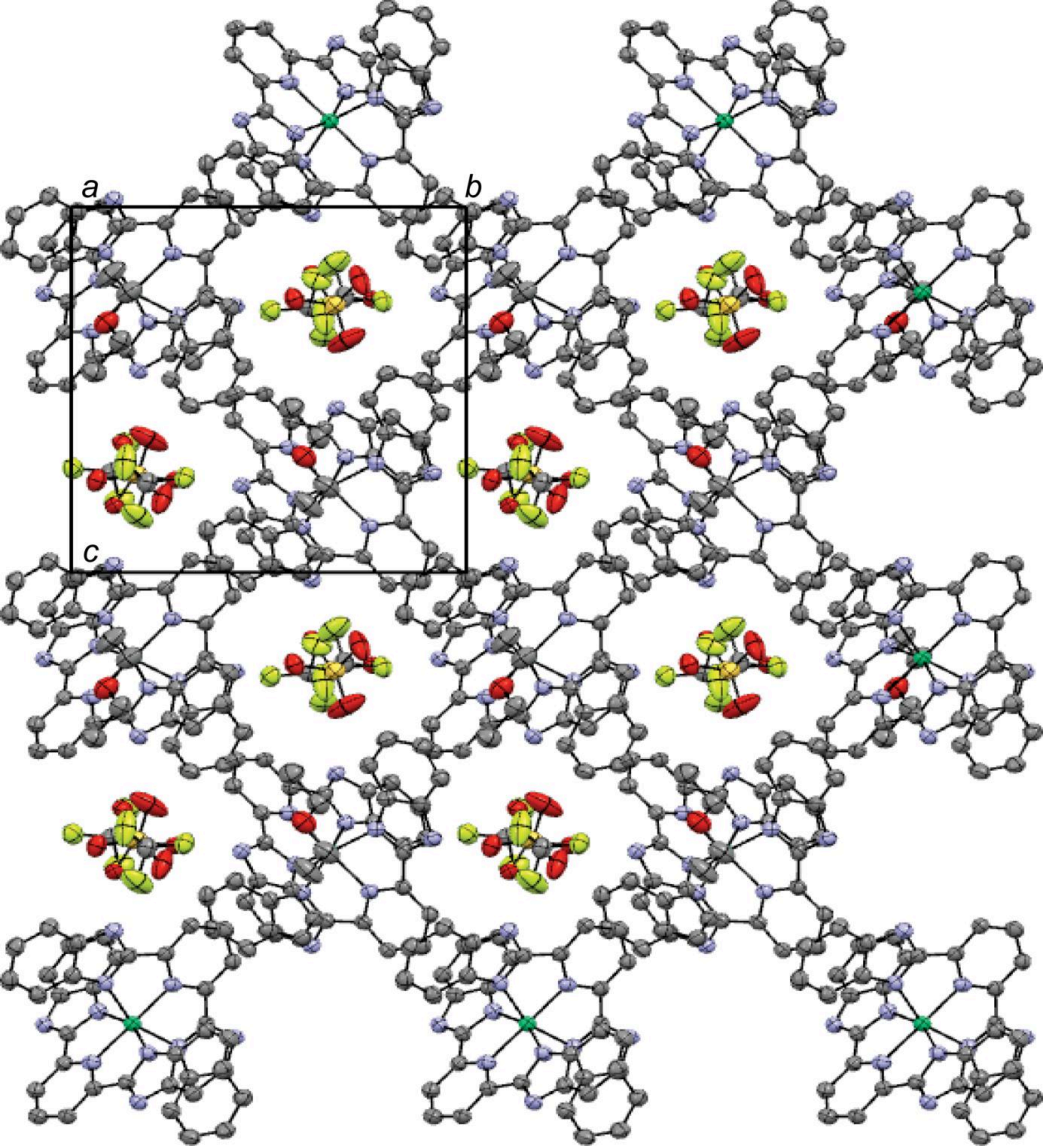
Perspective view of the crystal packing of the title complex approximately along the *a*-axis direction. H atoms are omitted for clarity.

The packing diagram reveals the stacking of the asymmetric units in columns aligned along the *a*-axis direction, creating a porous supra­molecular structure with the tri­fluoro­methane­sulfonate ions occupying the voids in the structure (Fig. 2 ▸). Several hydrogen bonds between the tri­fluoro­methane­sulfonate oxygen atoms and hydrogen atoms in the dication contribute to this arrangement (Table 2 ▸). No other directional supra­molecular inter­actions are present in the crystal packing of the title compound.

## Synthesis and crystallization

The title complex was prepared by adding Ag(CF_3_SO_3_) (0.216 g, 0.840 mmol) to an aceto­nitrile suspension (60 ml) of NiCl_2_·6H_2_O (0.100 g, 0.420 mmol). The mixture was heated, with stirring, at 323 K for 2 h and then filtered using a PTFE syringe filter to remove the precipitated AgCl. 2,6-Bis(2-benzimidazol­yl)pyridine (0.130 g, 0.841 mmol) was added to the resulting solution and then heated at 323 K to reduce the volume of the solution to 10 ml. X-ray diffraction quality crystals of the title complex were obtained by vapor diffusion of diethyl ether over the resulting concentrated aceto­nitrile solution.

## Refinement

Crystal data, data collection and structure refinement details are summarized in Table 3 ▸. The structure was refined as a two-component twin with a refined BASF value of 0.4104 (13).

## Supplementary Material

Crystal structure: contains datablock(s) I. DOI: 10.1107/S2414314624000889/vm4063sup1.cif

Structure factors: contains datablock(s) I. DOI: 10.1107/S2414314624000889/vm4063Isup2.hkl

Supporting information file. DOI: 10.1107/S2414314624000889/vm4063Isup3.mol

CCDC reference: 2303151

Additional supporting information:  crystallographic information; 3D view; checkCIF report

## Figures and Tables

**Table 1:** Selected geometric parameters (Å, °)

Ni1—N9	2.130 (7)	Ni1—N1	2.114 (6)
Ni1—N8	2.017 (6)	Ni1—N4	2.123 (7)
Ni1—N6	2.153 (6)	Ni1—N3	2.028 (6)
			
N9—Ni1—N6	155.3 (2)	N1—Ni1—N4	155.8 (2)
N8—Ni1—N9	78.0 (2)	N4—Ni1—N9	92.8 (2)
N8—Ni1—N6	77.4 (2)	N4—Ni1—N6	93.1 (2)
N8—Ni1—N1	104.5 (2)	N3—Ni1—N9	101.2 (2)
N8—Ni1—N4	99.8 (2)	N3—Ni1—N6	103.5 (2)
N8—Ni1—N3	177.3 (3)	N3—Ni1—N1	78.1 (2)
N1—Ni1—N9	92.0 (2)	N3—Ni1—N4	77.7 (2)
N1—Ni1—N6	92.3 (2)		

**Table 2:** Hydrogen-bond geometry (Å, °)

*D*—H⋯*A*	*D*—H	H⋯*A*	*D*⋯*A*	*D*—H⋯*A*
N2—H2⋯O2	0.88	2.14	2.836 (8)	136
N7—H7⋯O4^i^	0.88	2.18	3.052 (11)	171
N5—H5⋯O4	0.88	2.31	2.922 (11)	127
N10—H10⋯O6^ii^	0.88	2.25	2.933 (9)	135
N10—H10⋯O3^iii^	0.88	2.41	3.032 (9)	128

Symmetry codes: (i) 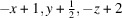 ; (ii) 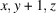 ; (iii) 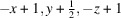 .

**Table 3:** Experimental details

Crystal data	
Chemical formula	[Ni(C_19_H_13_N_5_)_2_](CF_3_SO_3_)_2_·C_4_H_10_O
*M*_r_	1053.66
Crystal system, space group	Monoclinic, *P*2_1_
Temperature (K)	100
*a*, *b*, *c* (Å)	12.1089 (3), 13.3568 (3), 14.0513 (4)
β (°)	98.955 (3)
*V* (Å^3^)	2244.90 (10)
*Z*	2
Radiation type	Cu *K*α
μ (mm^−1^)	2.27
Crystal size (mm)	0.08 × 0.08 × 0.06

Data collection	
Diffractometer	XtaLAB Synergy, Dualflex, HyPix
Absorption correction	Gaussian (*CrysAlis PRO*; Rigaku OD, 2022 ▸)
*T*_min_, *T*_max_	0.014, 0.145
No. of measured, independent and observed [*I* > 2σ(*I*)] reflections	11492, 11492, 10931
(sin θ/λ)_max_ (Å^−1^)	0.630

Refinement	
*R*[*F* ^2^ > 2σ(*F* ^2^)], *wR*(*F* ^2^), *S*	0.058, 0.136, 1.07
No. of reflections	11492
No. of parameters	634
No. of restraints	1
H-atom treatment	H-atom parameters constrained
Δρ_max_, Δρ_min_ (e Å^−3^)	0.47, −0.28
Absolute structure	Classical Flack method preferred over Parsons because s.u. lower
Absolute structure parameter	0.00 (2)

Computer programs: *CrysAlis PRO* (Rigaku OD, 2022 ▸), *SHELXT* (Sheldrick, 2015*a* ▸), *SHELXL2018/3* (Sheldrick, 2015*b* ▸) and *OLEX2* (Dolomanov *et al.*, 2009 ▸).

## full crystallographic data

### Crystal data

**Table:**

[Ni(C_19_H_13_N_5_)_2_](CF_3_SO_3_)_2_·C_4_H_10_O	*F*(000) = 1080
*M**_r_* = 1053.66	*D*_x_ = 1.559 Mg m^−^^3^
Monoclinic, *P*2_1_	Cu *K*α radiation, λ = 1.54184 Å
*a* = 12.1089 (3) Å	Cell parameters from 12040 reflections
*b* = 13.3568 (3) Å	θ = 3.7–73.5°
*c* = 14.0513 (4) Å	µ = 2.27 mm^−^^1^
β = 98.955 (3)°	*T* = 100 K
*V* = 2244.90 (10) Å^3^	Plate, clear colourless
*Z* = 2	0.08 × 0.08 × 0.06 mm

### Data collection

**Table:**

XtaLAB Synergy, Dualflex, HyPix diffractometer	11492 measured reflections
Radiation source: micro-focus sealed X-ray tube, PhotonJet (Cu) X-ray Source	11492 independent reflections
Mirror monochromator	10931 reflections with *I* > 2σ(*I*)
Detector resolution: 10.0000 pixels mm^-1^	θ_max_ = 76.2°, θ_min_ = 3.2°
ω scans	*h* = −15→14
Absorption correction: gaussian (CrysAlisPro; Rigaku OD, 2022)	*k* = −16→16
*T*_min_ = 0.014, *T*_max_ = 0.145	*l* = −17→17

### Refinement

**Table:**

Refinement on *F*^2^	Hydrogen site location: inferred from neighbouring sites
Least-squares matrix: full	H-atom parameters constrained
*R*[*F*^2^ > 2σ(*F*^2^)] = 0.058	*w* = 1/[σ^2^(*F*_o_^2^) + (0.050*P*)^2^ + 2.5*P*] where *P* = (*F*_o_^2^ + 2*F*_c_^2^)/3
*wR*(*F*^2^) = 0.136	(Δ/σ)_max_ < 0.001
*S* = 1.07	Δρ_max_ = 0.47 e Å^−^^3^
11492 reflections	Δρ_min_ = −0.28 e Å^−^^3^
634 parameters	Absolute structure: Classical Flack method preferred over Parsons because s.u. lower
1 restraint	Absolute structure parameter: 0.00 (2)
Primary atom site location: dual	

### Special details

**Table:**

Geometry. All esds (except the esd in the dihedral angle between two l.s. planes) are estimated using the full covariance matrix. The cell esds are taken into account individually in the estimation of esds in distances, angles and torsion angles; correlations between esds in cell parameters are only used when they are defined by crystal symmetry. An approximate (isotropic) treatment of cell esds is used for estimating esds involving l.s. planes.
Refinement. Refined as a 2-component twin.

### Fractional atomic coordinates and isotropic or equivalent isotropic displacement parameters (Å^2^)

**Table:**

	*x*	*y*	*z*	*U*_iso_*/*U*_eq_	
Ni1	0.50632 (10)	0.65435 (10)	0.76555 (8)	0.0407 (3)	
S2	0.12531 (15)	0.16821 (17)	0.73086 (13)	0.0483 (5)	
S1	0.56101 (15)	0.65885 (17)	0.26452 (11)	0.0444 (4)	
F3	0.4341 (4)	0.5038 (4)	0.2844 (4)	0.0600 (13)	
F5	−0.0436 (4)	0.2869 (4)	0.7412 (4)	0.0591 (12)	
F1	0.3856 (4)	0.6378 (5)	0.3527 (4)	0.0776 (17)	
O6	0.1414 (5)	0.0643 (5)	0.7476 (5)	0.0582 (15)	
N2	0.7063 (5)	0.6697 (5)	0.5513 (4)	0.0432 (14)	
H2	0.718066	0.642731	0.496620	0.052*	
N7	0.7275 (5)	0.6077 (5)	1.0201 (4)	0.0423 (14)	
H7	0.755640	0.636859	1.074683	0.051*	
F2	0.3508 (4)	0.6213 (6)	0.1983 (4)	0.0850 (19)	
F4	−0.0233 (5)	0.1658 (6)	0.8446 (5)	0.104 (3)	
O1	0.5401 (5)	0.7636 (5)	0.2534 (4)	0.0581 (15)	
O3	0.5901 (5)	0.6091 (4)	0.1816 (4)	0.0490 (13)	
N9	0.3994 (5)	0.7710 (5)	0.7021 (4)	0.0415 (14)	
N8	0.5189 (5)	0.7559 (5)	0.8729 (4)	0.0398 (13)	
O2	0.6271 (5)	0.6297 (5)	0.3543 (4)	0.0552 (15)	
N5	0.2661 (5)	0.4311 (5)	0.7626 (4)	0.0433 (14)	
H5	0.239063	0.376827	0.731903	0.052*	
N6	0.6242 (5)	0.5851 (5)	0.8767 (4)	0.0399 (14)	
N1	0.6335 (5)	0.6949 (5)	0.6854 (4)	0.0404 (14)	
O4	0.1889 (6)	0.2296 (6)	0.7993 (6)	0.092 (3)	
C14	0.2991 (6)	0.5575 (6)	0.8663 (5)	0.0428 (16)	
C13	0.3487 (6)	0.4907 (6)	0.7390 (5)	0.0404 (16)	
N4	0.3704 (5)	0.5680 (5)	0.7993 (4)	0.0425 (14)	
N10	0.3142 (5)	0.9148 (5)	0.7293 (4)	0.0413 (14)	
H10	0.294992	0.968015	0.759795	0.050*	
F6	−0.0903 (4)	0.1373 (5)	0.6991 (6)	0.110 (3)	
C33	0.3290 (6)	0.7978 (6)	0.6186 (5)	0.0428 (17)	
O7	1.0079 (6)	0.5886 (6)	0.6835 (5)	0.0737 (19)	
C31	0.4549 (6)	0.8398 (6)	0.8615 (5)	0.0397 (16)	
O5	0.1204 (7)	0.1962 (8)	0.6346 (6)	0.119 (4)	
C9	0.5365 (6)	0.4890 (6)	0.5125 (5)	0.0424 (17)	
H9	0.581500	0.492939	0.462963	0.051*	
C16	0.2085 (7)	0.5847 (7)	1.0009 (6)	0.052 (2)	
H16	0.198024	0.623517	1.055447	0.062*	
N3	0.4863 (5)	0.5508 (5)	0.6588 (4)	0.0429 (14)	
C30	0.4568 (6)	0.9081 (6)	0.9357 (5)	0.0414 (16)	
H30	0.413634	0.967738	0.926984	0.050*	
C19	0.2326 (6)	0.4718 (6)	0.8436 (5)	0.0440 (17)	
C20	0.6452 (6)	0.6446 (6)	0.9524 (5)	0.0403 (16)	
C29	0.5237 (7)	0.8875 (7)	1.0234 (6)	0.0475 (19)	
H29	0.524204	0.931933	1.076295	0.057*	
C2	0.7176 (6)	0.7643 (6)	0.6827 (5)	0.0419 (16)	
C15	0.2872 (6)	0.6146 (7)	0.9465 (5)	0.0469 (18)	
H15	0.331897	0.672334	0.963139	0.056*	
C32	0.3890 (6)	0.8421 (6)	0.7656 (5)	0.0398 (16)	
C26	0.7587 (6)	0.5168 (6)	0.9879 (6)	0.0450 (18)	
C3	0.7568 (6)	0.8402 (6)	0.7474 (6)	0.0474 (18)	
H3	0.725107	0.850940	0.804352	0.057*	
C25	0.8349 (7)	0.4442 (7)	1.0284 (6)	0.0483 (19)	
H25	0.879128	0.453552	1.089815	0.058*	
C11	0.3895 (6)	0.4101 (6)	0.5839 (5)	0.0438 (17)	
H11	0.333945	0.359801	0.583438	0.053*	
C34	0.3121 (7)	0.7529 (7)	0.5277 (5)	0.0477 (18)	
H34	0.349060	0.692578	0.515659	0.057*	
C21	0.6940 (6)	0.5033 (6)	0.8973 (6)	0.0448 (18)	
C10	0.4549 (7)	0.4157 (7)	0.5118 (5)	0.0491 (19)	
H10A	0.443901	0.368480	0.460650	0.059*	
C37	0.1997 (7)	0.9344 (7)	0.5629 (6)	0.0501 (19)	
H37	0.161663	0.994138	0.575059	0.060*	
C28	0.5898 (6)	0.8014 (6)	1.0335 (5)	0.0409 (16)	
H28	0.636807	0.786987	1.092531	0.049*	
C38	0.2748 (7)	0.8874 (6)	0.6343 (5)	0.0430 (17)	
C12	0.4081 (6)	0.4811 (6)	0.6571 (5)	0.0419 (17)	
C36	0.1839 (7)	0.8892 (7)	0.4742 (6)	0.051 (2)	
H36	0.133862	0.918901	0.423180	0.061*	
C6	0.8510 (7)	0.8095 (6)	0.5749 (6)	0.0473 (18)	
H6	0.882512	0.799691	0.517750	0.057*	
C7	0.7627 (6)	0.7498 (6)	0.5978 (5)	0.0431 (17)	
C5	0.8887 (7)	0.8831 (7)	0.6409 (6)	0.053 (2)	
H5A	0.948210	0.925049	0.628445	0.064*	
C17	0.1428 (7)	0.4987 (7)	0.9789 (6)	0.0501 (19)	
H17	0.090028	0.480291	1.019249	0.060*	
C1	0.6294 (6)	0.6404 (6)	0.6058 (5)	0.0404 (16)	
C27	0.5854 (6)	0.7388 (6)	0.9567 (5)	0.0399 (16)	
C4	0.8430 (7)	0.8989 (7)	0.7259 (6)	0.051 (2)	
H4	0.871959	0.950831	0.768849	0.061*	
C42	1.0015 (8)	0.6461 (11)	0.7661 (7)	0.074 (3)	
H42A	0.983211	0.716507	0.748110	0.088*	
H42B	1.073803	0.644660	0.810065	0.088*	
C35	0.2398 (7)	0.7998 (7)	0.4563 (6)	0.056 (2)	
H35	0.227103	0.771517	0.393543	0.067*	
C24	0.8430 (7)	0.3586 (7)	0.9757 (6)	0.052 (2)	
H24	0.894924	0.308332	1.000978	0.063*	
C39	0.4253 (7)	0.6035 (8)	0.2760 (6)	0.053 (2)	
C18	0.1535 (7)	0.4407 (7)	0.9002 (6)	0.0474 (18)	
H18	0.109387	0.382328	0.884968	0.057*	
C23	0.7766 (7)	0.3433 (7)	0.8854 (7)	0.054 (2)	
H23	0.783922	0.282640	0.851552	0.065*	
C40	−0.0173 (7)	0.1910 (7)	0.7536 (7)	0.055 (2)	
C22	0.7008 (7)	0.4149 (7)	0.8449 (6)	0.0485 (19)	
H22	0.655383	0.404490	0.784214	0.058*	
C8	0.5495 (6)	0.5563 (6)	0.5889 (5)	0.0427 (17)	
C43	1.0938 (7)	0.6242 (8)	0.6328 (7)	0.061 (2)	
H43A	1.166897	0.623792	0.675634	0.074*	
H43B	1.077298	0.693607	0.610174	0.074*	
C44	1.0970 (9)	0.5566 (9)	0.5495 (8)	0.076 (3)	
H44A	1.026320	0.561634	0.505107	0.114*	
H44B	1.107936	0.487496	0.572428	0.114*	
H44C	1.158890	0.576087	0.516031	0.114*	
C41	0.9092 (9)	0.5999 (10)	0.8152 (9)	0.093 (4)	
H41A	0.837036	0.607265	0.773246	0.140*	
H41B	0.906508	0.634276	0.876452	0.140*	
H41C	0.924961	0.528713	0.827395	0.140*	

### Atomic displacement parameters (Å^2^)

**Table:**

	*U*^11^	*U*^22^	*U*^33^	*U*^12^	*U*^13^	*U*^23^
Ni1	0.0473 (6)	0.0379 (7)	0.0380 (6)	0.0014 (6)	0.0095 (5)	−0.0013 (6)
S2	0.0466 (9)	0.0497 (12)	0.0492 (10)	0.0037 (9)	0.0090 (7)	0.0070 (10)
S1	0.0537 (9)	0.0425 (10)	0.0377 (8)	−0.0029 (10)	0.0098 (7)	−0.0020 (9)
F3	0.060 (3)	0.055 (3)	0.067 (3)	−0.015 (2)	0.019 (2)	−0.004 (3)
F5	0.058 (3)	0.049 (3)	0.069 (3)	0.007 (2)	0.008 (2)	−0.004 (3)
F1	0.079 (3)	0.087 (5)	0.076 (3)	0.004 (3)	0.041 (3)	−0.008 (3)
O6	0.048 (3)	0.051 (4)	0.077 (4)	0.000 (3)	0.015 (3)	−0.011 (3)
N2	0.054 (3)	0.039 (4)	0.039 (3)	0.005 (3)	0.013 (2)	−0.005 (3)
N7	0.044 (3)	0.041 (4)	0.041 (3)	0.000 (3)	0.005 (2)	−0.001 (3)
F2	0.058 (3)	0.113 (6)	0.079 (4)	0.003 (3)	−0.003 (3)	0.015 (4)
F4	0.103 (4)	0.125 (6)	0.101 (4)	0.049 (5)	0.063 (4)	0.047 (5)
O1	0.084 (4)	0.048 (4)	0.046 (3)	0.005 (3)	0.021 (3)	0.001 (3)
O3	0.065 (3)	0.042 (3)	0.041 (3)	−0.003 (3)	0.014 (2)	0.001 (2)
N9	0.047 (3)	0.038 (4)	0.039 (3)	−0.001 (3)	0.006 (3)	0.000 (3)
N8	0.044 (3)	0.042 (4)	0.035 (3)	0.002 (3)	0.012 (2)	0.000 (3)
O2	0.067 (3)	0.056 (4)	0.040 (3)	−0.013 (3)	0.003 (2)	−0.003 (3)
N5	0.054 (4)	0.037 (4)	0.039 (3)	−0.003 (3)	0.009 (3)	−0.004 (3)
N6	0.043 (3)	0.036 (4)	0.041 (3)	0.006 (3)	0.011 (2)	−0.001 (3)
N1	0.043 (3)	0.040 (4)	0.040 (3)	−0.002 (3)	0.012 (2)	−0.004 (3)
O4	0.067 (4)	0.056 (5)	0.139 (7)	0.016 (4)	−0.028 (4)	−0.031 (5)
C14	0.049 (4)	0.036 (4)	0.044 (4)	−0.001 (3)	0.009 (3)	0.002 (3)
C13	0.046 (4)	0.037 (4)	0.039 (4)	0.004 (3)	0.008 (3)	−0.002 (3)
N4	0.048 (3)	0.039 (4)	0.041 (3)	0.001 (3)	0.007 (3)	−0.002 (3)
N10	0.048 (3)	0.044 (4)	0.034 (3)	0.004 (3)	0.010 (3)	−0.002 (3)
F6	0.053 (3)	0.059 (4)	0.207 (8)	0.000 (3)	−0.018 (4)	−0.017 (5)
C33	0.044 (4)	0.046 (5)	0.038 (4)	0.000 (3)	0.005 (3)	−0.001 (3)
O7	0.067 (4)	0.077 (5)	0.079 (4)	−0.001 (4)	0.015 (3)	0.017 (4)
C31	0.044 (4)	0.036 (4)	0.040 (4)	0.000 (3)	0.013 (3)	0.002 (3)
O5	0.093 (5)	0.179 (11)	0.094 (5)	0.061 (6)	0.048 (4)	0.076 (6)
C9	0.051 (4)	0.040 (5)	0.037 (4)	−0.001 (4)	0.012 (3)	−0.003 (3)
C16	0.060 (5)	0.053 (5)	0.045 (4)	−0.003 (4)	0.015 (3)	−0.010 (4)
N3	0.045 (3)	0.049 (4)	0.035 (3)	0.002 (3)	0.008 (2)	−0.001 (3)
C30	0.046 (4)	0.033 (4)	0.046 (4)	0.003 (3)	0.011 (3)	0.001 (3)
C19	0.051 (4)	0.044 (5)	0.037 (4)	0.001 (4)	0.009 (3)	−0.001 (3)
C20	0.044 (3)	0.039 (4)	0.038 (3)	0.002 (3)	0.008 (3)	0.003 (3)
C29	0.054 (5)	0.044 (5)	0.046 (4)	−0.002 (4)	0.015 (3)	−0.004 (4)
C2	0.046 (4)	0.038 (4)	0.041 (4)	0.006 (3)	0.005 (3)	0.000 (3)
C15	0.053 (4)	0.044 (5)	0.045 (4)	−0.005 (4)	0.009 (3)	−0.005 (4)
C32	0.046 (4)	0.035 (4)	0.040 (4)	0.005 (3)	0.012 (3)	−0.001 (3)
C26	0.050 (4)	0.037 (4)	0.050 (4)	0.002 (3)	0.014 (3)	−0.003 (4)
C3	0.051 (4)	0.045 (5)	0.047 (4)	0.001 (4)	0.011 (3)	−0.005 (4)
C25	0.045 (4)	0.047 (5)	0.052 (4)	0.000 (4)	0.004 (3)	0.006 (4)
C11	0.046 (4)	0.041 (4)	0.044 (4)	−0.001 (3)	0.006 (3)	0.001 (4)
C34	0.056 (4)	0.045 (5)	0.042 (4)	0.004 (4)	0.007 (3)	−0.001 (4)
C21	0.047 (4)	0.041 (4)	0.049 (4)	0.005 (3)	0.016 (3)	0.004 (4)
C10	0.062 (5)	0.047 (5)	0.038 (4)	−0.002 (4)	0.007 (3)	−0.005 (4)
C37	0.054 (4)	0.049 (5)	0.048 (4)	0.006 (4)	0.009 (3)	0.003 (4)
C28	0.047 (4)	0.038 (4)	0.038 (4)	−0.002 (3)	0.008 (3)	0.002 (3)
C38	0.052 (4)	0.041 (5)	0.036 (4)	−0.004 (4)	0.008 (3)	0.000 (3)
C12	0.051 (4)	0.037 (4)	0.037 (4)	−0.003 (3)	0.004 (3)	0.006 (3)
C36	0.054 (4)	0.054 (6)	0.042 (4)	0.006 (4)	−0.001 (3)	0.002 (4)
C6	0.054 (4)	0.045 (5)	0.044 (4)	0.004 (4)	0.011 (3)	0.004 (4)
C7	0.048 (4)	0.040 (4)	0.042 (4)	0.004 (3)	0.006 (3)	−0.002 (3)
C5	0.050 (4)	0.052 (5)	0.058 (5)	−0.007 (4)	0.013 (4)	0.002 (4)
C17	0.055 (4)	0.052 (5)	0.047 (4)	−0.003 (4)	0.017 (4)	−0.003 (4)
C1	0.046 (3)	0.039 (5)	0.036 (3)	0.004 (3)	0.008 (3)	0.000 (3)
C27	0.046 (4)	0.038 (4)	0.037 (4)	0.000 (3)	0.009 (3)	0.006 (3)
C4	0.056 (4)	0.048 (5)	0.049 (4)	−0.005 (4)	0.004 (3)	−0.008 (4)
C42	0.064 (5)	0.086 (8)	0.073 (6)	0.013 (6)	0.019 (4)	0.023 (6)
C35	0.064 (5)	0.061 (6)	0.041 (4)	0.002 (4)	0.002 (4)	−0.010 (4)
C24	0.053 (4)	0.045 (5)	0.060 (5)	0.009 (4)	0.010 (4)	0.005 (4)
C39	0.053 (4)	0.060 (6)	0.047 (4)	0.000 (4)	0.007 (3)	0.002 (4)
C18	0.055 (4)	0.043 (5)	0.046 (4)	0.001 (4)	0.012 (3)	0.003 (4)
C23	0.061 (5)	0.038 (5)	0.067 (6)	0.004 (4)	0.021 (4)	0.000 (4)
C40	0.047 (4)	0.051 (6)	0.066 (5)	0.004 (4)	0.009 (4)	−0.003 (4)
C22	0.051 (4)	0.046 (5)	0.051 (4)	0.002 (4)	0.014 (3)	−0.006 (4)
C8	0.053 (4)	0.039 (4)	0.036 (3)	0.002 (4)	0.007 (3)	0.001 (3)
C43	0.055 (4)	0.062 (6)	0.070 (6)	0.000 (4)	0.017 (4)	0.009 (5)
C44	0.075 (6)	0.080 (8)	0.072 (6)	−0.007 (6)	0.005 (5)	−0.008 (6)
C41	0.074 (6)	0.104 (10)	0.106 (9)	0.029 (7)	0.029 (6)	0.056 (8)

### Geometric parameters (Å, º)

**Table:**

Ni1—N9	2.130 (7)	N3—C8	1.338 (9)
Ni1—N8	2.017 (6)	C30—H30	0.9500
Ni1—N6	2.153 (6)	C30—C29	1.393 (11)
Ni1—N1	2.114 (6)	C19—C18	1.401 (11)
Ni1—N4	2.123 (7)	C20—C27	1.457 (11)
Ni1—N3	2.028 (6)	C29—H29	0.9500
S2—O6	1.416 (7)	C29—C28	1.396 (11)
S2—O4	1.400 (7)	C2—C3	1.395 (11)
S2—O5	1.396 (7)	C2—C7	1.401 (10)
S2—C40	1.829 (8)	C15—H15	0.9500
S1—O1	1.426 (6)	C26—C25	1.398 (11)
S1—O3	1.432 (6)	C26—C21	1.398 (11)
S1—O2	1.438 (6)	C3—H3	0.9500
S1—C39	1.832 (9)	C3—C4	1.377 (12)
F3—C39	1.339 (11)	C25—H25	0.9500
F5—C40	1.325 (10)	C25—C24	1.374 (12)
F1—C39	1.328 (10)	C11—H11	0.9500
N2—H2	0.8800	C11—C10	1.382 (10)
N2—C7	1.378 (10)	C11—C12	1.392 (11)
N2—C1	1.352 (9)	C34—H34	0.9500
N7—H7	0.8800	C34—C35	1.375 (11)
N7—C20	1.359 (9)	C21—C22	1.401 (11)
N7—C26	1.369 (10)	C10—H10A	0.9500
F2—C39	1.325 (9)	C37—H37	0.9500
F4—C40	1.335 (10)	C37—C38	1.394 (11)
N9—C33	1.385 (9)	C37—C36	1.371 (11)
N9—C32	1.323 (9)	C28—H28	0.9500
N8—C31	1.358 (10)	C28—C27	1.361 (10)
N8—C27	1.339 (9)	C36—H36	0.9500
N5—H5	0.8800	C36—C35	1.414 (12)
N5—C13	1.359 (10)	C6—H6	0.9500
N5—C19	1.378 (9)	C6—C7	1.411 (11)
N6—C20	1.321 (9)	C6—C5	1.379 (12)
N6—C21	1.384 (10)	C5—H5A	0.9500
N1—C2	1.383 (10)	C5—C4	1.408 (12)
N1—C1	1.329 (9)	C17—H17	0.9500
C14—N4	1.381 (9)	C17—C18	1.372 (11)
C14—C19	1.408 (11)	C1—C8	1.478 (11)
C14—C15	1.386 (11)	C4—H4	0.9500
C13—N4	1.334 (10)	C42—H42A	0.9900
C13—C12	1.454 (10)	C42—H42B	0.9900
N10—H10	0.8800	C42—C41	1.532 (14)
N10—C32	1.370 (10)	C35—H35	0.9500
N10—C38	1.395 (9)	C24—H24	0.9500
F6—C40	1.293 (11)	C24—C23	1.408 (12)
C33—C34	1.397 (10)	C18—H18	0.9500
C33—C38	1.398 (11)	C23—H23	0.9500
O7—C42	1.404 (13)	C23—C22	1.385 (12)
O7—C43	1.431 (10)	C22—H22	0.9500
C31—C30	1.382 (10)	C43—H43A	0.9900
C31—C32	1.456 (10)	C43—H43B	0.9900
C9—H9	0.9500	C43—C44	1.483 (14)
C9—C10	1.390 (11)	C44—H44A	0.9800
C9—C8	1.390 (10)	C44—H44B	0.9800
C16—H16	0.9500	C44—H44C	0.9800
C16—C15	1.371 (11)	C41—H41A	0.9800
C16—C17	1.404 (12)	C41—H41B	0.9800
N3—C12	1.325 (10)	C41—H41C	0.9800
			
N9—Ni1—N6	155.3 (2)	C24—C25—C26	117.1 (8)
N8—Ni1—N9	78.0 (2)	C24—C25—H25	121.4
N8—Ni1—N6	77.4 (2)	C10—C11—H11	121.3
N8—Ni1—N1	104.5 (2)	C10—C11—C12	117.4 (7)
N8—Ni1—N4	99.8 (2)	C12—C11—H11	121.3
N8—Ni1—N3	177.3 (3)	C33—C34—H34	121.4
N1—Ni1—N9	92.0 (2)	C35—C34—C33	117.2 (8)
N1—Ni1—N6	92.3 (2)	C35—C34—H34	121.4
N1—Ni1—N4	155.8 (2)	N6—C21—C26	109.0 (7)
N4—Ni1—N9	92.8 (2)	N6—C21—C22	129.7 (7)
N4—Ni1—N6	93.1 (2)	C26—C21—C22	121.1 (8)
N3—Ni1—N9	101.2 (2)	C9—C10—H10A	119.3
N3—Ni1—N6	103.5 (2)	C11—C10—C9	121.4 (8)
N3—Ni1—N1	78.1 (2)	C11—C10—H10A	119.3
N3—Ni1—N4	77.7 (2)	C38—C37—H37	122.0
O6—S2—C40	104.1 (4)	C36—C37—H37	122.0
O4—S2—O6	114.4 (4)	C36—C37—C38	116.0 (8)
O4—S2—C40	102.5 (5)	C29—C28—H28	120.8
O5—S2—O6	114.0 (5)	C27—C28—C29	118.4 (7)
O5—S2—O4	116.5 (6)	C27—C28—H28	120.8
O5—S2—C40	103.0 (4)	N10—C38—C33	106.0 (7)
O1—S1—O3	115.4 (3)	C37—C38—N10	131.2 (8)
O1—S1—O2	115.3 (4)	C37—C38—C33	122.8 (7)
O1—S1—C39	105.0 (4)	N3—C12—C13	111.4 (7)
O3—S1—O2	114.5 (4)	N3—C12—C11	121.6 (7)
O3—S1—C39	102.8 (4)	C11—C12—C13	127.0 (7)
O2—S1—C39	101.4 (4)	C37—C36—H36	119.0
C7—N2—H2	126.6	C37—C36—C35	122.0 (8)
C1—N2—H2	126.6	C35—C36—H36	119.0
C1—N2—C7	106.9 (6)	C7—C6—H6	122.2
C20—N7—H7	126.3	C5—C6—H6	122.2
C20—N7—C26	107.4 (6)	C5—C6—C7	115.7 (7)
C26—N7—H7	126.3	N2—C7—C2	106.3 (7)
C33—N9—Ni1	142.9 (5)	N2—C7—C6	132.0 (7)
C32—N9—Ni1	111.0 (5)	C2—C7—C6	121.7 (7)
C32—N9—C33	105.9 (6)	C6—C5—H5A	118.5
C31—N8—Ni1	119.5 (5)	C6—C5—C4	123.0 (8)
C27—N8—Ni1	120.5 (5)	C4—C5—H5A	118.5
C27—N8—C31	120.0 (6)	C16—C17—H17	119.4
C13—N5—H5	126.5	C18—C17—C16	121.2 (8)
C13—N5—C19	107.0 (7)	C18—C17—H17	119.4
C19—N5—H5	126.5	N2—C1—C8	128.3 (6)
C20—N6—Ni1	110.4 (5)	N1—C1—N2	112.3 (7)
C20—N6—C21	105.7 (6)	N1—C1—C8	119.3 (6)
C21—N6—Ni1	143.6 (5)	N8—C27—C20	110.3 (7)
C2—N1—Ni1	141.9 (5)	N8—C27—C28	122.4 (7)
C1—N1—Ni1	111.9 (5)	C28—C27—C20	127.3 (7)
C1—N1—C2	105.9 (6)	C3—C4—C5	120.9 (8)
N4—C14—C19	108.9 (7)	C3—C4—H4	119.6
N4—C14—C15	130.9 (7)	C5—C4—H4	119.6
C15—C14—C19	120.2 (7)	O7—C42—H42A	110.3
N5—C13—C12	127.9 (7)	O7—C42—H42B	110.3
N4—C13—N5	112.2 (6)	O7—C42—C41	106.9 (10)
N4—C13—C12	119.8 (7)	H42A—C42—H42B	108.6
C14—N4—Ni1	142.7 (5)	C41—C42—H42A	110.3
C13—N4—Ni1	111.2 (5)	C41—C42—H42B	110.3
C13—N4—C14	105.8 (6)	C34—C35—C36	121.5 (8)
C32—N10—H10	126.9	C34—C35—H35	119.2
C32—N10—C38	106.1 (6)	C36—C35—H35	119.2
C38—N10—H10	126.9	C25—C24—H24	119.0
N9—C33—C34	130.2 (7)	C25—C24—C23	121.9 (8)
N9—C33—C38	109.3 (6)	C23—C24—H24	119.0
C34—C33—C38	120.3 (7)	F3—C39—S1	110.5 (6)
C42—O7—C43	111.6 (8)	F1—C39—S1	112.0 (6)
N8—C31—C30	120.9 (7)	F1—C39—F3	107.8 (7)
N8—C31—C32	110.3 (6)	F2—C39—S1	111.1 (6)
C30—C31—C32	128.8 (7)	F2—C39—F3	106.6 (7)
C10—C9—H9	121.4	F2—C39—F1	108.6 (7)
C8—C9—H9	121.4	C19—C18—H18	121.7
C8—C9—C10	117.1 (7)	C17—C18—C19	116.7 (8)
C15—C16—H16	118.8	C17—C18—H18	121.7
C15—C16—C17	122.4 (7)	C24—C23—H23	119.4
C17—C16—H16	118.8	C22—C23—C24	121.2 (8)
C12—N3—Ni1	119.7 (5)	C22—C23—H23	119.4
C12—N3—C8	121.0 (7)	F5—C40—S2	110.6 (6)
C8—N3—Ni1	119.3 (5)	F5—C40—F4	108.7 (8)
C31—C30—H30	120.8	F4—C40—S2	108.6 (6)
C31—C30—C29	118.4 (7)	F6—C40—S2	112.4 (7)
C29—C30—H30	120.8	F6—C40—F5	109.3 (7)
N5—C19—C14	106.1 (7)	F6—C40—F4	107.0 (9)
N5—C19—C18	131.9 (8)	C21—C22—H22	121.5
C18—C19—C14	122.0 (7)	C23—C22—C21	117.1 (8)
N7—C20—C27	126.8 (7)	C23—C22—H22	121.5
N6—C20—N7	112.0 (7)	C9—C8—C1	127.5 (7)
N6—C20—C27	121.2 (6)	N3—C8—C9	121.5 (7)
C30—C29—H29	120.1	N3—C8—C1	111.0 (6)
C30—C29—C28	119.8 (8)	O7—C43—H43A	110.2
C28—C29—H29	120.1	O7—C43—H43B	110.2
N1—C2—C3	130.3 (7)	O7—C43—C44	107.5 (9)
N1—C2—C7	108.5 (7)	H43A—C43—H43B	108.5
C3—C2—C7	121.2 (7)	C44—C43—H43A	110.2
C14—C15—H15	121.2	C44—C43—H43B	110.2
C16—C15—C14	117.5 (8)	C43—C44—H44A	109.5
C16—C15—H15	121.2	C43—C44—H44B	109.5
N9—C32—N10	112.6 (7)	C43—C44—H44C	109.5
N9—C32—C31	120.9 (7)	H44A—C44—H44B	109.5
N10—C32—C31	126.5 (7)	H44A—C44—H44C	109.5
N7—C26—C25	132.8 (8)	H44B—C44—H44C	109.5
N7—C26—C21	105.8 (7)	C42—C41—H41A	109.5
C25—C26—C21	121.5 (8)	C42—C41—H41B	109.5
C2—C3—H3	121.2	C42—C41—H41C	109.5
C4—C3—C2	117.6 (8)	H41A—C41—H41B	109.5
C4—C3—H3	121.2	H41A—C41—H41C	109.5
C26—C25—H25	121.4	H41B—C41—H41C	109.5
			
Ni1—N9—C33—C34	−10.4 (14)	C16—C17—C18—C19	−0.1 (13)
Ni1—N9—C33—C38	173.4 (6)	C30—C31—C32—N9	178.4 (8)
Ni1—N9—C32—N10	−175.5 (5)	C30—C31—C32—N10	0.2 (13)
Ni1—N9—C32—C31	6.2 (9)	C30—C29—C28—C27	−1.1 (11)
Ni1—N8—C31—C30	176.3 (5)	C19—N5—C13—N4	−0.4 (9)
Ni1—N8—C31—C32	−2.8 (8)	C19—N5—C13—C12	−177.6 (7)
Ni1—N8—C27—C20	3.0 (8)	C19—C14—N4—Ni1	−174.3 (6)
Ni1—N8—C27—C28	−174.8 (6)	C19—C14—N4—C13	−0.7 (9)
Ni1—N6—C20—N7	−173.9 (5)	C19—C14—C15—C16	−0.6 (12)
Ni1—N6—C20—C27	4.9 (8)	C20—N7—C26—C25	−177.0 (8)
Ni1—N6—C21—C26	172.7 (6)	C20—N7—C26—C21	1.8 (8)
Ni1—N6—C21—C22	−11.3 (14)	C20—N6—C21—C26	−0.7 (8)
Ni1—N1—C2—C3	5.2 (14)	C20—N6—C21—C22	175.3 (8)
Ni1—N1—C2—C7	−173.5 (6)	C29—C28—C27—N8	−1.1 (11)
Ni1—N1—C1—N2	176.4 (5)	C29—C28—C27—C20	−178.5 (7)
Ni1—N1—C1—C8	−6.0 (8)	C2—N1—C1—N2	0.3 (8)
Ni1—N3—C12—C13	−1.6 (8)	C2—N1—C1—C8	177.8 (6)
Ni1—N3—C12—C11	178.3 (6)	C2—C3—C4—C5	0.6 (13)
Ni1—N3—C8—C9	−177.9 (6)	C15—C14—N4—Ni1	4.6 (15)
Ni1—N3—C8—C1	1.9 (8)	C15—C14—N4—C13	178.2 (8)
O6—S2—C40—F5	179.4 (6)	C15—C14—C19—N5	−178.6 (7)
O6—S2—C40—F4	60.2 (8)	C15—C14—C19—C18	−0.4 (12)
O6—S2—C40—F6	−58.1 (8)	C15—C16—C17—C18	−0.9 (14)
N2—C1—C8—C9	−0.1 (13)	C32—N9—C33—C34	175.8 (8)
N2—C1—C8—N3	−179.9 (7)	C32—N9—C33—C38	−0.4 (8)
N7—C20—C27—N8	173.2 (7)	C32—N10—C38—C33	0.2 (8)
N7—C20—C27—C28	−9.1 (12)	C32—N10—C38—C37	−178.5 (9)
N7—C26—C25—C24	178.2 (8)	C32—C31—C30—C29	177.1 (7)
N7—C26—C21—N6	−0.7 (8)	C26—N7—C20—N6	−2.4 (8)
N7—C26—C21—C22	−177.1 (7)	C26—N7—C20—C27	178.9 (7)
O1—S1—C39—F3	178.4 (5)	C26—C25—C24—C23	−1.0 (13)
O1—S1—C39—F1	−61.4 (7)	C26—C21—C22—C23	−1.9 (12)
O1—S1—C39—F2	60.3 (7)	C3—C2—C7—N2	179.8 (7)
O3—S1—C39—F3	57.4 (6)	C3—C2—C7—C6	1.5 (12)
O3—S1—C39—F1	177.5 (6)	C25—C26—C21—N6	178.3 (7)
O3—S1—C39—F2	−60.8 (7)	C25—C26—C21—C22	1.8 (12)
N9—C33—C34—C35	−176.8 (8)	C25—C24—C23—C22	0.9 (13)
N9—C33—C38—N10	0.1 (8)	C34—C33—C38—N10	−176.5 (7)
N9—C33—C38—C37	179.0 (7)	C34—C33—C38—C37	2.3 (12)
N8—C31—C30—C29	−1.8 (11)	C21—N6—C20—N7	1.9 (8)
N8—C31—C32—N9	−2.6 (10)	C21—N6—C20—C27	−179.3 (7)
N8—C31—C32—N10	179.2 (7)	C21—C26—C25—C24	−0.4 (12)
O2—S1—C39—F3	−61.3 (6)	C10—C9—C8—N3	0.0 (11)
O2—S1—C39—F1	58.9 (7)	C10—C9—C8—C1	−179.8 (7)
O2—S1—C39—F2	−179.4 (7)	C10—C11—C12—C13	179.0 (8)
N5—C13—N4—Ni1	176.5 (5)	C10—C11—C12—N3	−0.8 (11)
N5—C13—N4—C14	0.7 (9)	C37—C36—C35—C34	0.8 (14)
N5—C13—C12—N3	−177.8 (7)	C38—N10—C32—N9	−0.5 (9)
N5—C13—C12—C11	2.3 (13)	C38—N10—C32—C31	177.8 (7)
N5—C19—C18—C17	178.4 (8)	C38—C33—C34—C35	−0.9 (12)
N6—C20—C27—N8	−5.3 (10)	C38—C37—C36—C35	0.5 (13)
N6—C20—C27—C28	172.3 (7)	C12—C13—N4—Ni1	−6.1 (9)
N6—C21—C22—C23	−177.5 (8)	C12—C13—N4—C14	178.1 (7)
N1—C2—C3—C4	−179.9 (8)	C12—N3—C8—C9	−0.5 (11)
N1—C2—C7—N2	−1.4 (8)	C12—N3—C8—C1	179.3 (7)
N1—C2—C7—C6	−179.7 (7)	C12—C11—C10—C9	0.3 (12)
N1—C1—C8—C9	−177.2 (7)	C36—C37—C38—N10	176.4 (8)
N1—C1—C8—N3	3.0 (10)	C36—C37—C38—C33	−2.0 (12)
O4—S2—C40—F5	60.0 (7)	C6—C5—C4—C3	0.1 (14)
O4—S2—C40—F4	−59.2 (8)	C7—N2—C1—N1	−1.2 (8)
O4—S2—C40—F6	−177.5 (8)	C7—N2—C1—C8	−178.4 (7)
C14—C19—C18—C17	0.7 (12)	C7—C2—C3—C4	−1.4 (12)
C13—N5—C19—C14	0.0 (9)	C7—C6—C5—C4	−0.1 (12)
C13—N5—C19—C18	−178.0 (9)	C5—C6—C7—N2	−178.5 (8)
N4—C14—C19—N5	0.5 (9)	C5—C6—C7—C2	−0.7 (11)
N4—C14—C19—C18	178.7 (7)	C17—C16—C15—C14	1.2 (13)
N4—C14—C15—C16	−179.4 (8)	C1—N2—C7—C2	1.5 (8)
N4—C13—C12—N3	5.2 (10)	C1—N2—C7—C6	179.6 (8)
N4—C13—C12—C11	−174.7 (7)	C1—N1—C2—C3	179.4 (8)
C33—N9—C32—N10	0.6 (9)	C1—N1—C2—C7	0.7 (8)
C33—N9—C32—C31	−177.8 (7)	C27—N8—C31—C30	−0.4 (11)
C33—C34—C35—C36	−0.6 (13)	C27—N8—C31—C32	−179.5 (6)
C31—N8—C27—C20	179.7 (6)	C42—O7—C43—C44	177.8 (8)
C31—N8—C27—C28	1.9 (11)	C24—C23—C22—C21	0.6 (12)
C31—C30—C29—C28	2.5 (11)	C8—C9—C10—C11	0.1 (12)
O5—S2—C40—F5	−61.3 (8)	C8—N3—C12—C13	−178.9 (7)
O5—S2—C40—F4	179.4 (8)	C8—N3—C12—C11	1.0 (11)
O5—S2—C40—F6	61.2 (9)	C43—O7—C42—C41	−178.8 (8)

### Hydrogen-bond geometry (Å, º)

**Table:**

*D*—H···*A*	*D*—H	H···*A*	*D*···*A*	*D*—H···*A*
N2—H2···O2	0.88	2.14	2.836 (8)	136
N7—H7···O4^i^	0.88	2.18	3.052 (11)	171
N5—H5···O4	0.88	2.31	2.922 (11)	127
N10—H10···O6^ii^	0.88	2.25	2.933 (9)	135
N10—H10···O3^iii^	0.88	2.41	3.032 (9)	128

Symmetry codes: (i) −*x*+1, *y*+1/2, −*z*+2; (ii) *x*, *y*+1, *z*; (iii) −*x*+1, *y*+1/2, −*z*+1.
